# Prediction of liquid-phase separation proteins using Siamese network with feature fusion

**DOI:** 10.1093/bib/bbaf393

**Published:** 2025-08-06

**Authors:** Ye-Hong Yang, Qun Liu, Jiang-Feng Liu, Jun-Tao Yang

**Affiliations:** School of Basic Medicine, Nanchang Medical College, No. 689, Hui Ren Da Dao, Xiaolan Economic Development Zone, Nanchang, 330006, China; State Key Laboratory of Common Mechanism Research for Major Diseases, Department of Biochemistry and Molecular Biology, Institute of Basic Medical Sciences, Chinese Academy of Medical Sciences & Peking Union Medical College, No. 5, Dongdan 3, Dongcheng District Municipality of Beijing, Beijing, 100005, China; School of Basic Medicine, Nanchang Medical College, No. 689, Hui Ren Da Dao, Xiaolan Economic Development Zone, Nanchang, 330006, China; State Key Laboratory of Common Mechanism Research for Major Diseases, Department of Biochemistry and Molecular Biology, Institute of Basic Medical Sciences, Chinese Academy of Medical Sciences & Peking Union Medical College, No. 5, Dongdan 3, Dongcheng District Municipality of Beijing, Beijing, 100005, China; School of Basic Medicine, Nanchang Medical College, No. 689, Hui Ren Da Dao, Xiaolan Economic Development Zone, Nanchang, 330006, China; State Key Laboratory of Common Mechanism Research for Major Diseases, Department of Biochemistry and Molecular Biology, Institute of Basic Medical Sciences, Chinese Academy of Medical Sciences & Peking Union Medical College, No. 5, Dongdan 3, Dongcheng District Municipality of Beijing, Beijing, 100005, China

**Keywords:** liquid–liquid phase separation, PPI network embedding, graph embedding, feature fusion, deep learning

## Abstract

Liquid–liquid phase separation (LLPS) is a common and important phenomenon where biomolecules form dynamic, membrane-less condensates through multivalent interactions, spontaneously separating into distinct concentration-dense and dilute phases. Research has shown that LLPS is associated with a wide range of cellular functional regulation. In this work, we establish a feature fusion framework based on a Siamese network for the prediction of LLPS proteins, which can integrate automatically extracted features from the protein itself and the protein–protein interaction (PPI) networks, and achieve good accuracy even in small sample sets. We used two representative graph embedding methods, Node2vec and DeepNF, to extract the embedding features of PPI networks and compared the impact of the two methods on model performance at different feature lengths. Our work provides a way for integrating multivalent interactions between proteins that drive LLPS, as well as a flexible framework for the fusion of different types of protein features, not only for LLPS prediction but also for other downstream prediction tasks. All relevant materials can be found at https://github.com/ispotato/SiameseNetwork_LLPS.

## Introduction

Liquid–liquid phase separation (LLPS) is a biological molecular process that supports the formation of membraneless organelles within living cells, which is also one of the important biophysical mechanisms mediating the formation of protein-rich biomolecular condensates by large molecules such as proteins and nucleic acids [1–3]. LLPS is closely related to various cellular events, such as transcriptional control, autophagy, and chromatin formation [4–7], and the abnormal liquid-phase behavior of proteins is related to the occurrence and development of neurodegenerative diseases, metabolic diseases, and cancer [8–11]. Therefore, studying the features and functions of liquid-phase-separated proteins is of great significance for further understanding the driving mechanisms and processes of protein liquid-phase behavior [12, 13]. The experiments have shown that multivalent interactions between protein domains can form liquid-phase-separated droplets, which are spherical structures that can flow, fuse, and recover after fluorescence bleaching [14–16]. Meanwhile, in addition to domains with regular structures, some intrinsically disordered regions (IDRs) or low-complexity domains can also trigger multivalent interactions between proteins, leading to liquid-phase separation [14]. However, the complexity of protein sequences and the diversity of conformations pose significant challenges for experimental validation, thus requiring the development of LLPS prediction algorithm models to assist in experimental discoveries to promote research on LLPS and its mechanisms.

Currently, some studies have established LLPS prediction tools that use experimentally validated LLPS proteins based on specific protein features such as amino acid composition, structural disorder, domains, and physicochemical properties to train machine learning models [17–20]. However, these models still used manually designed features, which may result in limitations in the expression of biological information, such as the complex features that are difficult to manually design, as well as the related factors of liquid-phase separation that we have not yet recognized. With the development of deep learning technology, the new generation of LLPS prediction models has begun to use natural language processing (NLP) methods and artificial intelligence models to automatically extract protein sequence and structure features [21–24]. DeePhase used engineer features (including the molecular weight, amino acid composition, hydrophobicity, and the Shannon entropy) and a pretrained word2vec model (3 g of protein sequence as words and created 200-dimensional embedding features) to establish the machine learning classifier separately, and finally combined them to provide an average prediction score [21]. PSPredictor combined evolutionary word2vec with machine learning algorithms and obtained good accuracy [22]. PredLLPS_PSSM integrates the word embedding features with the position-specific score matrix (PSSM), which is considered to contain the evolutionary information of protein sequences [23]. PSPire utilizes the protein PDB (Protein Data Bank, the database is http://www.rcsb.org) structures predicted by Alphafold2 to calculate the IDRs and to determine the structural superficial regions (SSUP) and then constructs the XGBoost classifier using these features [25].

Although these studies have made great progress in automatic feature extraction, there are still shortcomings. These models all use word2vec, which ignores the positional information of amino acids in protein sequences and is limited by the size of the window, which makes it difficult to represent features from the perspective of the complete sequence [26–28]. Another issue with these LLPS predictors is that they are based solely on the features of individual proteins, which may miss out the LLPS proteins driven by complex modular interaction domains or motifs and other multivalent interactions, especially the proteins with low IDR contents, and cannot undergo liquid-phase separation alone [29]. Integrating the information of protein–protein interaction (PPI) networks into LLPS models may be helpful [24, 29]. PSPSHunter merged the above individual protein features with four generic properties of PPI networks, which comprised degree, betweenness, clustering coefficient, and average neighbor degree [24]. However, due to the heterogeneity of biological networks and their varying sparsity and noise levels, automatically extracting the features from PPI networks remains challenging. One possible approach is a graph embedding method such as Node2vec, which takes the adjacency matrix of the PPI network as input and encodes each node in the network as a vector [30]. Another method is the autoencoder network, which, unlike the shallow and linear techniques of Node2vec, can capture complex and highly nonlinear PPI network structural features [31]. These graph embedding methods have not yet been applied to LLPS prediction models, nor have they been compared with the performance after fusing different features.

The large language models (LLMs) based on the Transformer architecture have made significant progress in the field of NLP [32, 33]. The multi-head attention mechanism of Transformer allows the model to learn the relationships between all amino acids in each protein sequence from multiple different subspaces, without being limited by long distances; thus, the LLM pretrained on a massive set of protein sequences may exhibit a form of emergent behavior capable of capturing latent structural, functional, and evolutionary information of proteins [34–36]. Studies have used protein features obtained from LLM pretrained models in downstream tasks such as protein 3D structure prediction and protein subcellular localization, and the results have shown that their performance is better than manually designed features or features obtained from smaller feature extraction models [36–38].

In this work, our goal is to establish a neural network model suitable for small sample sets that can integrate features automatically extracted from the protein itself and the PPI network to achieve LLPS prediction. Firstly, we use the extracted features from the representative large-scale pretraining model ESM2 (Evolutionary Scale Modeling) [34] as the features of the protein itself. Then, we chose two graph embedding methods, namely, deep autoencoder and Node2vec, to extract more comprehensive and computable features from the protein PPI network, thereby integrating the external correlation information of the protein into the LLPS prediction model. Finally, to address the issue of limited sample size, we designed a flexible framework based on a multi-channel Siamese network that achieves good performance at small sample sizes by comparing the similarity between input sample pairs [39, 40].

## Materials and methods

### Benchmark dataset

To train the deep learning model, we rigorously curated both positive and negative datasets to ensure representativeness and minimize bias.

For the positive dataset (LLPS proteins), we primarily collected LLPS proteins from four authoritative databases, including LLPSDB [41], DrLLPS [42], PhaSepDB [43], and PhaSePro [44]. Finally, we obtained 859 LLPS proteins spanning 47 species, which were composed of scaffold proteins and self-LLPS proteins. Among these, 452 were human LLPS proteins, ensuring relevance to human biology. For negative samples (non-LLPS proteins), we adopted PDB-derived negative samples that have been widely used in published LLPS prediction models [20, 21, 45], as structured proteins are empirically unlikely to undergo homotypic LLPS under physiological conditions [21]. Specifically, we first extracted fully structured amino acid sequences (excluding any disordered residues) and used a conservative cutoff value of 30% to eliminate the remaining sequences. Finally, we obtained 1560 proteins belonging to 460 species as the negative sample set (Supplementary materials).

To obtain PPI information related to LLPS proteins and compare the impact of different databases on the specificity and coverage of LLPS prediction models, we selected two representative PPI databases: STRING (Search Tool for the Retrieval of Interacting Genes/Proteins, the database is https://cn.string-db.org/) [46] and BioPlex [47].

The PPI data in the STRING database primarily originate from experimentally validated and computationally predicted interactions, with each interaction assigned a confidence score to evaluate its reliability. To minimize noise in the PPI data, we applied a relatively stringent interaction score threshold (>0.2) to filter the PPI data from STRING. This threshold excludes low-confidence predictions (based on STRING benchmark tests showing >80% precision at this threshold) while maintaining a reasonable sample size and data coverage [46]. We collected PPI datasets for all species in the above LLPS sample set from the STRING database [46] and ultimately obtained PPI networks for 206 species. Then, we screened out the interaction pairs involving LLPS proteins with interaction scores >0.2 from these PPI datasets, respectively [46]. Finally, in the LLPS sample set mentioned above, there are 1235 proteins with PPI-related information, including 724 positive proteins and 511 negative proteins (Supplementary materials).

To construct training pairs for the Siamese network, we developed an enhanced sampling algorithm that improves upon the original fixed-offset approach through three key modifications: (i) multi-round sampling (num_repeats = 3) to increase data diversity, (ii) dynamic positive pair generation with random offsets (1 to max_offset = 5) instead of fixed intervals, and (iii) randomized negative pair selection by sampling from arbitrary different classes. The label assignment follows the standard contrastive learning convention: 1 indicates positive pairs (samples from the same class), and 0 indicates negative pairs (samples from different classes). From the STRING database, we selected 1022 samples with PPI features (511 positive and 511 negative cases). These samples were initially divided into an independent validation set containing 206 samples (20%), with the remaining 816 samples subjected to 10-fold cross-validation. Ultimately, we obtained 4272 training pairs, 360 testing pairs, and 1176 independent validation pairs (see Supplementary Materials).

To analyze the impact of PPI data incompleteness on the model’s generalization performance and prediction results, we also selected another representative database, BioPlex, which contains only high-confidence PPI data validated by affinity purification mass spectrometry (average confidence >0.93 [47]) but is limited to the human proteome [47]. We integrated results from all human cell lines in this database and ultimately obtained PPI information for 361 LLPS proteins, including 252 positive proteins and 109 negative proteins (Supplementary Materials). Using the same sampling method, from the BioPlex database, we obtained 660 training pairs, 204 testing pairs, and 216 independent validation pairs (see Supplementary Materials).

### Protein feature representation

With the development of large language models, pretrained protein language models (PLMs) have also made significant progress [32, 33]. By treating protein sequences as sentences and amino acid residues as words, PLM models based on transformer architecture can effectively capture and express complex contextual features of protein sequences, atomic-level structural features, and evolutionary information contained in proteins [34–38]. Representative PLM models include ESM-2, ESM-1b, ESM-1v, ProtBert, and Prot-T5-XL; among them, ESM-2 performs better than previous ESM models and other PLMs in a range of structural prediction [34, 38]. Using ESM2 to automatically extract protein features avoids the tedious workload and potential bias caused by manual feature design and has achieved good results in some downstream prediction tasks [36, 37]. Considering the size of the LLPS dataset, we selected models with parameters of 8M, 35M, 150M, and 650M, corresponding to embedding feature dimensions of 320, 480, 640, and 1280, respectively [34].

The ProtTrans family of models (including ProtBert and ProtT5-XL) represents another widely adopted PLM framework. These models employ strategies similar to natural language processing models (BERT and T5 architectures) and undergo pretraining on ultra-large-scale datasets [48, 49]. Through sequence reconstruction–based pretraining strategies, these models have demonstrated outstanding performance in various functional predictions and remote homology detection tasks [48, 49]. To compare the impact of different large language models on LLPS prediction performance, we utilized the ProtBert submodel from the ProtTrans series. We extracted features for LLPS sample proteins under two different pretraining dataset conditions: UniRef100 (remove redundant UniProt reference sequences) and BFD (Big Fantastic Database). In both cases, the resulting embedded feature dimensions were 1024. The features of LLPS protein samples extracted using ProtTrans are detailed in the Supplementary Materials.

### Protein–protein interaction network feature representation

In the process of liquid-phase separation, multivalent interactions between molecules are also an important driving force, especially for some proteins that cannot be separated in liquid phase alone [28]. One possible approach is to extract the external related features from protein interaction networks; however, due to the complexity of biological networks, manually defined features are difficult to accurately express this information [23, 28]. In the field of knowledge graphs, there are some graph-embedding methods that can be used to map the nodes and edges of the graph into a low-dimensional vector space, so that similar nodes in the original graph also maintain similar distances in the vector space [29, 30]. This not only avoids the potential bias caused by manually designed features but also facilitates the use of these embedding vectors as input features for subsequent deep learning models.

To evaluate the impact of different graph-embedding methods on feature extraction and predictive performance for LLPS, we employed four representative graph embedding models: Node2vec, DeepNF, Line2vec, and Struct2vec to extract features from the PPI network of LLPS proteins. To investigate the relationship between feature dimension size and model prediction accuracy, we tested three distinct feature lengths (64, 128, and 256 dimensions). The following section details these four graph-embedding approaches.

#### Network embedding based on Node2vec

Node2vec is a semi-supervised algorithm used to learn continuous feature representations (embeddings) of nodes in a network, which can adapt to different types of graph data for effectively capturing graph structures and generating high-quality node embeddings [30]. It employs a biased random walk strategy to explore network neighborhoods, balancing breadth-first (BFS) and depth-first (DFS) sampling through two tunable parameters *p* and *q*. The objective function maximizes the log-probability of observing network neighborhoods *N_s_* (*u*) for each node *u*:


(1) \begin{equation*} \underset{f}{\max}\sum_{u\in V}\mathit{\log}{P}_r\left({N}_s(u)|f(u)\right) \end{equation*}

where *f*(*u*) ∈ *R^d^* denotes the *d*-dimensional embedding of node *u*, and *V* is the set of nodes. The walk transition probability between nodes *v* and *x* is given by:


(2) \begin{equation*} \mathrm{P}\left({c}_i=x|{c}_{i-1}=v\right)=\left\{\begin{array}{@{}c}\frac{\pi_{vx}}{Z},\mathrm{if}\left(\mathrm{v},\mathrm{x}\right)\in \mathrm{E}\\{}0,\mathrm{otherwise}\end{array}\right. \end{equation*}

where π_vx_ is the unnormalized transition probability (adjusted by *p* and *q*), and *Z* is a normalization constant. For PPI networks, this method captures both local topological features and global functional relationships between proteins.

#### Network embedding based on deep autoencoder

DeepNF is a deep autoencoder–based network fusion method that integrates heterogeneous networks into compact low-dimensional feature representations through a multi-layer nonlinear DNN architecture [31]. This approach enables DeepNF to learn richer network representations while demonstrating robustness to noisy links and corrupted data [31]. The method consists of two key steps:

Step 1: Network Representation via Random Walk with Restarts (RWR) and PPMI:

For each PPI network with adjacency matrix A^j^, we first compute a probabilistic co-occurrence matrix R^j^ using RWR:


(3) \begin{equation*} {P}_i^t=\alpha{P}_i^{\left(t-1\right)}\hat{A}+\left(1-\alpha \right){P}_i^0 \end{equation*}

where $\hat{A} $ is the row-normalized adjacency matrix, α is the restart probability (set to 0.98), and *P_i_^t^* represents the probability distribution of reaching other proteins from protein *i* after *t* steps. The final node representation *r_i_* is obtained by summing over *T* steps:


(4) \begin{equation*} {r}_i=\sum_{t=1}^T{p}_i^t\ \left(i=1,2,\dots, n\right) \end{equation*}

where *T* is the total number of random walk steps. Then, we calculate a vector representation of protein nodes by constructing a Positive Pointwise Mutual Information (PPMI) matrix as follows:


(5) \begin{equation*} {X}_{ij}=\mathit{\max}\left(0,{\mathit{\log}}_2\left(\frac{R_{ij}\sum_i\sum_j{R}_{ij}}{\sum_i{R}_{ij}\sum_j{R}_{ij}}\right)\right)\ \left(i,j=1,2,\dots, n\right) \end{equation*}

where ${X}_{ij}$ is the co-occurrence probability between nodes *i* and *j*. Through the PPMI matrix $\mathrm{X}\in{R}^{n\times n}$, we achieve the optimal approximation of the PPI network.

Step 2: Multimodal Deep Autoencoder (MDA) for Feature Fusion:

The PPMI matrices are fed into an MDA to learn a shared low-dimensional embedding:


(6) \begin{equation*} {H}_{encode}=\delta \left({W}_{encode}X+{B}_{encode}\right) \end{equation*}

where ${W}_{encode}\in{R}^{d\times n}$ and ${B}_{encode}\in{R}^{d\times n}$ are weight and bias matrices, respectively， and $\mathrm{\delta}$ is a sigmoid activation function. The model reconstructs the input PPMI matrices while minimizing the binary cross-entropy loss:


(7) \begin{equation*} L\left(\theta \right)=\sum_{j=1}^N\left({X}^j\mathit{\log}{\hat{X}}^j+\left(1-{X}^j\right)\log \left(1-{\hat{X}}^j\right)\right) \end{equation*}

#### Other network-embedding methods

##### Line2vec (large-scale information network embedding)

Line2vec is a graph embedding method designed to preserve both first-order and second-order proximity in large-scale networks. It optimizes two objective functions:

(1) First-order proximity captures direct edges between nodes by minimizing:


(8) \begin{equation*} {L}_1=-\sum_{\left(i,j\right)\in E}{w}_{i,j}\log \left(\delta \left({u}_i^T{v}_j\right)\right) \end{equation*}

where *u* and *v* are embeddings of nodes *i* and *j*, *w* is the edge weight, and $\delta$ is the sigmoid activation function.

(2) Second-order proximity considers shared neighbors via:


(9) \begin{equation*} {L}_2=-\sum_{\left(i,j\right)\in E}{w}_{i,j}\log \frac{\exp \left({u}_j^T{v}_i\right)}{\sum_{k=1}^{\mid V\mid}\exp \left({u}_k^T{v}_i\right)} \end{equation*}

The advantage of Line2vec is its high computational efficiency for large networks and the ability to simulate local and global structures clearly.

### Struct2vec

Struct2vec focuses on structural similarity between nodes, independent of their network distance. It constructs a multi-layer graph where each layer encodes hierarchical structural similarity, then uses random walks to generate context:


(10) \begin{equation*} {P}_{struct}\left(j|i\right)\propto \exp \left(- dist\left({R}_{i,}{R}_j\right)\right) \end{equation*}

where *R_i_* and *R_j_* are structural fingerprints (e.g. degree sequences) of nodes *i* and *j*, and dist function measures structural distance. The advantage of this algorithm is that it can capture functional similarity even without direct interaction and is robust to network noise.

### Liquid–liquid-phase separation model based on Siamese network

**Figure 1:**
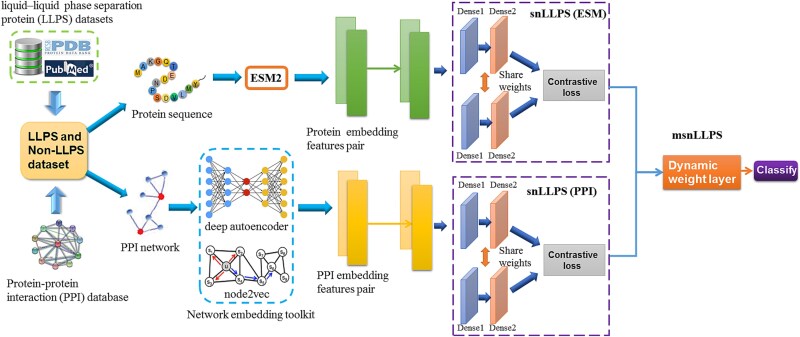
The framework of the Siamese network-based LLPS prediction models.

A Siamese network is a supervised learning model specifically designed for deep metric learning, consisting of two or more identical networks, whose core function is to evaluate the similarity between two inputs, and has been applied in fields such as facial recognition, speech recognition, and object tracking [39, 40]. The basic idea of Siamese networks is to input two samples into two subnetworks that share weights and parameters and then calculate their similarity by learning the representation vectors of the input data in each subnetwork, and finally perform classification or regression operations. Due to the fact that Siamese networks classify by comparing the similarity of input samples without relying on a large number of independent samples for training, they are suitable for small sample learning tasks. In this study, we designed a contrastive learning–based feature fusion framework that integrates ESM2 embedding feature pairs (processed by a Siamese network) with PPI graph embedding features (processed by another Siamese network) through a dynamic weighting layer. Through end-to-end learning, the framework adaptively adjusts feature weights to achieve a few-shot multi-feature fusion model for LLPS prediction. The overall architecture is illustrated in Fig. 1.

#### Single-channel Siamese network liquid–liquid phase separation prediction model

In this section, we constructed a single-channel Siamese network LLPS model, called snLLPS. As illustrated in Fig. 1, the model processes a pair of protein samples by feeding their feature vectors into two identical neural networks with shared parameters (Base network 1 and Base network 2 in Fig. 1). Then, by comparing the similarity of the output vectors of the two base networks to estimate whether the two sample proteins belong to the same classification, as shown in Fig. 1. The architecture description of the snLLPS model is as follows:

The input is two *d*-dimensional protein feature vectors (*x*_*a*,_  *x_b_*) from a pair (ESM2 embedding pair or PPI embedding pair), the base networks ${f}_{\theta }$ transforms each input into a latent representation:


(11) \begin{equation*} {h}_a={f}_{\theta}\left({x}_a\right),{h}_b={f}_{\theta}\left({x}_b\right) \end{equation*}

Then, the Euclidean distance between the outputs of the two base networks:


(12) \begin{equation*} \mathrm{D}\left({h}_a,{h}_b\right)={\left|\left|{h}_a-{h}_b\right|\right|}_2 \end{equation*}

The optimization objective of the snLLPS model is to minimize the following contrastive loss function on the training set:


(13) \begin{equation*} \arg \underset{\theta }{\min}\frac{1}{2N}\sum_{n=1}^N\left\{Y{D}_w^2+\left(1-Y\right){\left\{\max \left(m-{D}_w,0\right)\right\}}^2\right\} \end{equation*}

of which, if the input two proteins belong to the same category, then the value of *Y* is 0; otherwise, *Y* is 1. m is a threshold greater than 0. There is a margin indicating that dissimilar pairs exceeding this threshold will not result in loss.

#### Multi-channel Siamese network liquid–liquid phase separation prediction model

In this section, we extend the above Siamese network-based LLPS prediction model to incorporate multiple input features, named msnLLPS. As illustrated in Fig. 1, the model simultaneously processes two different feature vector pairs (ESM2 embedding pair and PPI embedding pair) of sample protein pairs, with each feature type corresponding to an independent Siamese subnetwork. Finally, the outputs from these two Siamese subnetworks are integrated through a dynamic weighting layer to predict whether the protein pair belongs to the same category. The architecture description of the model is as follows:

The input is two protein feature vectors (*x*^1^_*a*,_  *x*^1^*_b_*) with dimension *d*_1_ and feature vectors (*x*^2^_*a*,_  *x*^2^*_b_*) with dimension *d*_2_ from a pair, separate base network (${\mathrm{f}}_{\theta}^1,{f}_{\theta}^2$) project each feature type to latent spaces:


(14) \begin{equation*} {h}_a^1={f}_{\theta}^1\left({x}_a^1\right),{h}_b^1={f}_{\theta}^1\left({x}_b^1\right) \end{equation*}

(15) \begin{equation*} {h}_a^2={f}_{\theta}^2\left({x}_a^2\right),{h}_b^2={f}_{\theta}^2\left({x}_b^2\right) \end{equation*}

For dynamic weighted fusion, the Euclidean distance is still used to calculate the pairwise distance for each feature type:


(16) \begin{equation*} {D}_1\left({h}_a^1,{h}_b^1\right)={\left|\left|{h}_a^1-{h}_b^1\right|\right|}_2,{D}_2\left({h}_a^2,{h}_b^2\right)={\left|\left|{h}_a^2-{h}_b^2\right|\right|}_2 \end{equation*}

Finally, the distance is adaptively combined by the learnable dynamic weight layer:


(17) \begin{equation*} {d}_{final}={w}_1{d}_1+{w}_2{d}_{2,}\ where\ {w}_1+{w}_2=1 \end{equation*}

Then, the optimization objective of the msnLLPS model is to minimize the contrastive loss function:


(18) \begin{equation*} \mathrm{L}=\mathrm{y}{d}_{final}^2+\left(1-\mathrm{y}\right)\max{\left(\mathrm{m}-{d}_{final},0\right)}^2 \end{equation*}

### Model training and evaluation

To evaluate the performance of snLLPS and msnLLPS, we calculated the four different parameters: Accuracy (ACC), Precision, F1-score, and Matthews correlation coefficient (MCC). The four parameters are defined as follows:


$$\textrm{Precision}=\frac{TP}{TP+ FP} $$

$$\textrm{ACC}= \frac{TP+ TN}{TP+ FP+ TN+ FN} $$

$$ \textrm{F1}\hbox{-}\textrm{score}=\frac{2 TP}{2 TP+ FP+ FN} $$

$$\textrm{MCC}= \frac{TP\ast TN- FP\ast FN}{\sqrt{\left( TP+ FP\right)\ast \left( TP+ FN\right)\ast \left( TN+ FP\right)\ast \left( TN+ FN\right)}} $$

where TP, FP, TN, and FN are the numbers of true-positive samples, false-positive samples, true-negative samples, and false-negative samples, respectively.

## Results

### Comparison of Siamese network–based liquid–liquid phase separation models with different ESM2 feature lengths

**Figure 2:**
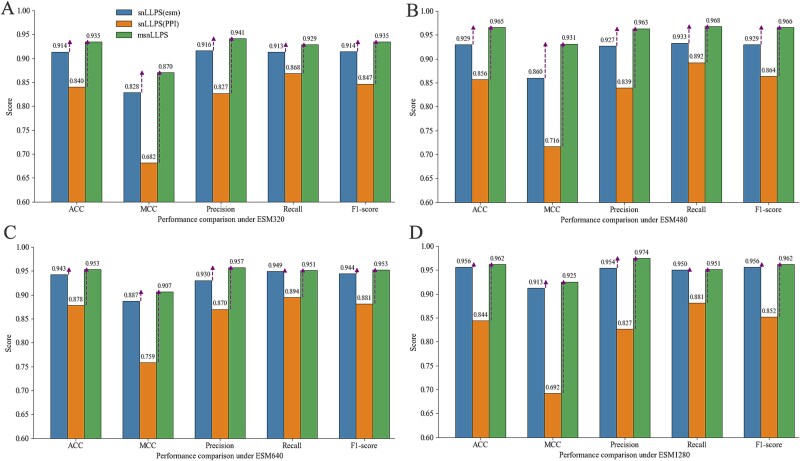
The test performance of snLLPS and msnLLPS on different ESM feature lengths. (A) The test performance of snLLPS and msnLLPS under ESM320. (B) The test performance of snLLPS and msnLLPS under ESM480. (C) The test performance of snLLPS and msnLLPS under ESM640. (D) The test performance of snLLPS and msnLLPS under ESM1280

In this section, we first compared the performance differences between single-channel Siamese network LLPS models (using either ESM2 embeddings or PPI network embeddings alone) and multi-channel Siamese network LLPS models (integrating both ESM2 and PPI network embeddings) across various evaluation metrics. Using Node2vec-derived PPI network embeddings with a fixed length of 64 as an example, we tested their combination with ESM2 embeddings of varying lengths (320, 480, 640, and 1280). From the STRING database, we extracted 1022 proteins with PPI embedding features (511 LLPS proteins and 511 non-LLPS proteins). Following the enhanced random pairing method described in the Materials and Methods section, we performed 10-fold cross-validation, generating 4272 training pairs and 360 testing pairs. The average results across the 10-fold validation are shown in Fig. 2.

Figure 2 demonstrates that: (i) All msnLLPS models outperformed their snLLPS counterparts, indicating that incorporating PPI network embeddings provides more informative features for prediction compared to using protein sequence features alone, thereby enhancing model performance (Fig. 2a-2d), and (ii) the msnLLPS model achieved optimal predictive performance when using ESM2 embeddings with 480 dimensions (Fig. 2b). While PPI embeddings improved performance across all configurations, the enhancement was most pronounced when combined with 480-dimensional ESM2 embeddings (Fig. 2b).

### Comparison of the Siamese network-based liquid–liquid phase separation models with different protein–protein interaction feature lengths

**Figure 3:**
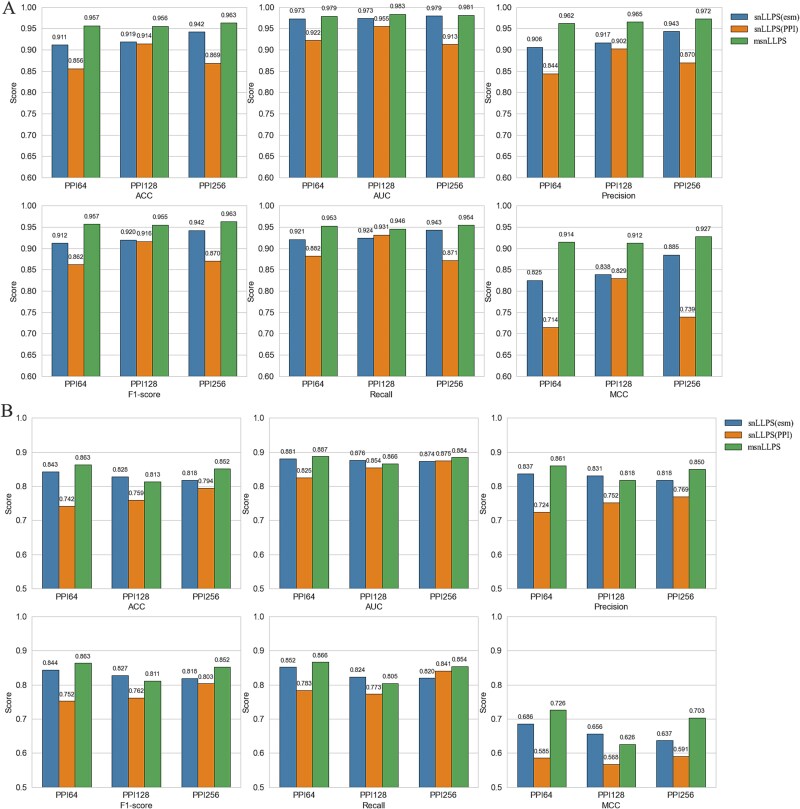
The performance of snLLPS and msnLLPS on different PPI feature lengths. (A) The test performance of snLLPS and msnLLPS under PPI64, PPI128 and PPI256. (B) The independent test performance of snLLPS and msnLLPS under PPI64, PPI128 and PPI256.

To systematically evaluate the impact of input embedding dimensions on Siamese network performance for LLPS prediction, we conducted comprehensive comparisons using ESM2 embeddings (fixed at 480 dimensions) combined with Node2vec-derived PPI embeddings of varying lengths (64, 128, and 256 dimensions). Using the same STRING database subset of 1022 proteins with PPI features (511 LLPS versus 511 non-LLPS), we allocated 80% for training/testing (yielding 4272 training pairs and 360 testing pairs through enhanced pairing) and 20% as an independent validation set (1176 pairs). Figure 3a and b presents the averaged 10-fold cross-validation test performance and independent validation results, respectively. Key findings reveal that: (i) for PPI-only snLLPS models, 128-dimensional embeddings achieved optimal predictive accuracy, while 256-dimensional embeddings showed superior generalization, and (ii) the msnLLPS model attained peak prediction performance with 256-dimensional PPI features but demonstrated best generalization with 64-dimensional features. These observations suggest that current PPI data may provide limited informational gain, as excessively complex feature combinations in msnLLPS could lead to overfitting.

### Comparison of the Siamese network-based liquid–liquid phase separation models with different graph-embedding methods

**Figure 4:**
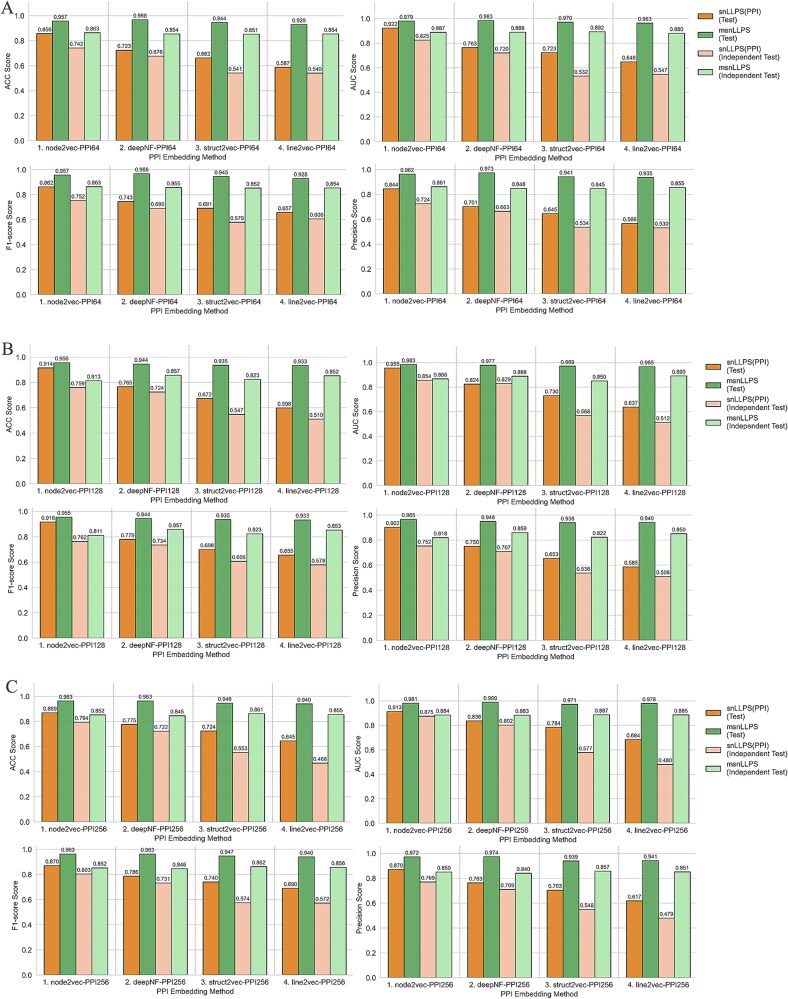
The performance of snLLPS and msnLLPS on different graph-embedding methods. (A) The performance of snLLPS and msnLLPS on different graph embedding methods with PPI64. (B) The performance of snLLPS and msnLLPS on different graph embedding methods with PPI128. (C) The performance of snLLPS and msnLLPS on graph embedding methods with PPI256.

We systematically evaluated the impact of different graph embedding methods on both snLLPS and msnLLPS models by comparing four representative techniques (Node2vec, DeepNF, Line2vec, and Struct2vec) under standardized conditions. Using the STRING database with fixed-length embeddings (ESM2: 480D; PPI: 64D) and identical test sets as previous experiments, Figure 4 presents the averaged 10-fold cross-validation and independent validation results. Key observations include: (i) for snLLPS models, Node2vec and DeepNF-derived PPI features significantly outperformed Line2vec and Struct2vec across ACC, AUC, F1-score, and Precision, with Node2vec showing particular advantage in capturing LLPS-relevant patterns. This performance disparity originates from fundamental methodological differences: Node2vec’s biased random walks and DeepNF’s nonlinear dimensionality reduction preserve functionally critical network neighborhoods at meso-scales (5–50 nodes), whereas Line2vec’s edge-centric approach and Struct2vec’s rigid graphlet isomorphism fail to retain essential biological context. (ii) While msnLLPS exhibited similar trends in embedding method efficacy, its performance degradation with suboptimal PPI features was markedly attenuated. This robustness stems from the dynamic weighting layer’s capacity to adaptively downweight less informative PPI features while prioritizing high-quality ESM2 embeddings, demonstrating the architecture’s resilience to imperfect auxiliary data.

### Comparison of the Siamese network-based liquid–liquid phase separation models with different protein–protein interaction databases

**Figure 5:**
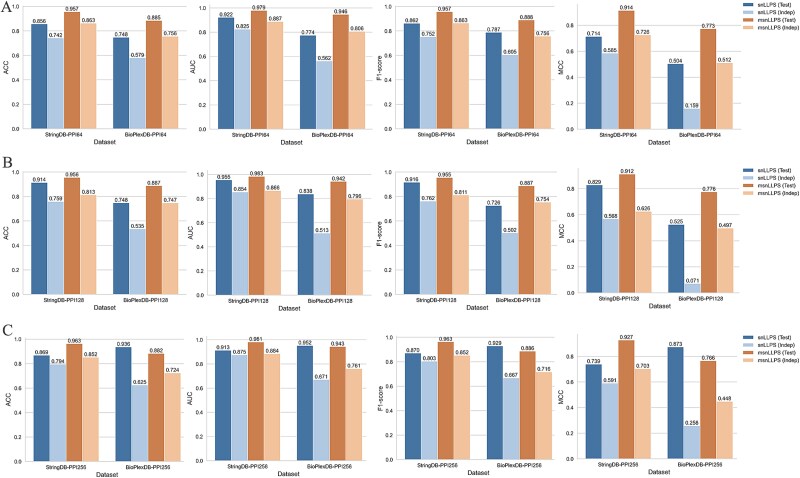
The performance of snLLPS and msnLLPS on different PPI databases. (A) The performance of snLLPS and msnLLPS on different PPI database with PPI64. (B) The performance of snLLPS and msnLLPS on different PPI database with PPI128. (B) The performance of snLLPS and msnLLPS on different PPI database with PPI256.

To evaluate the impact of PPI database selection on model performance, we compared two representative databases, STRING and BioPlex (detailed in Materials and Methods), using fixed-length embeddings (PPI: 64D; ESM2: 480D). The STRING dataset comprised 4272 training pairs, 360 testing pairs, and 1176 independent validation pairs, while BioPlex contained 660 training pairs, 204 testing pairs, and 216 validation pairs. As shown in Fig. 5, PPI features derived from STRING consistently outperformed those from BioPlex in both snLLPS and msnLLPS models across testing and independent validation metrics. Notably, BioPlex-based PPI features exhibited significant performance degradation in snLLPS’s independent validation (Fig. 5b), whereas msnLLPS’s dynamic weighting mechanism mitigated this decline, though its overall performance remained inferior to STRING-based models. Three primary factors explain this disparity: (i) species coverage: STRING includes interactions of 206 species in our benchmark, while BioPlex is restricted to human proteins; (2) data comprehensiveness: STRING integrates multi-evidence interactions (experimental, co-expression, and literature-derived) with confidence scoring, better capturing biologically relevant patterns than BioPlex’s binary Affinity Purification-Mass Spectrometry (AP-MS) data; and (3) network completeness: STRING incorporates computationally predicted interactions (e.g. phylogenetic profiles, gene fusion), covering more LLPS-related pathways than BioPlex’s experimentally detected interactions alone [47].

### Comparison of the Siamese network-based models with other liquid–liquid phase separation models

**Figure 6:**
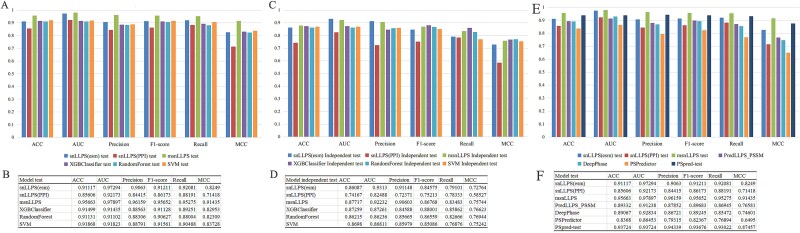
Comparing the performance of snLLPS and msnLLPS with other LLPS models. (A) The test performance of snLLPS, msnLLPS and the machine learning models. (B) The detailed data of test performance on snLLPS, msnLLPS and the machine learning models. (C) The independent test performance of snLLPS, msnLLPS and the machine learning models. (D) The detailed data of independent test on snLLPS, msnLLPS and the machine learning models. (E) The test performance of snLLPS, msnLLPS and other LLPS models. (F) The detailed data of snLLPS, msnLLPS and other LLPS models.

In this section, we first compared both snLLPS and msnLLPS with several classical traditional machine learning architectures. We consistently employed ESM2 embeddings with 480 dimensions (ESM480) and PPI network features extracted by Node2vec with 64 dimensions (PPI64), while maintaining the identical 10-fold cross-validation dataset configuration as described previously. For traditional machine learning models, the input features consisted solely of the ESM480 embeddings. The results of Fig. 6a demonstrate that the multi-feature fused msnLLPS model exhibits significant advantages in both test and independent validation sets, achieving superior test set accuracy (ACC: 0.957) and Matthews correlation coefficient (MCC: 0.914) that not only substantially outperform the single-feature snLLPS(ESM) (ACC: 0.911, MCC: 0.825) and snLLPS (PPI) (ACC: 0.856, MCC: 0.714) but also comprehensively exceed baseline models including XGBoost (ACC: 0.915), Random Forest (ACC: 0.911), and SVM (ACC: 0.919), the details are shown in the table of Fig. 6b. Particularly in independent validation scenarios, msnLLPS (MCC: 0.757) shows significantly smaller performance degradation compared to snLLPS (PPI) (ΔMCC: −0.129 versus −0.172), verifying the robust regulatory effect of the dynamic weighting layer against PPI feature noise, while its independent validation performance still stably leads all comparative models (e.g. 0.011 higher MCC than Random Forest), as shown in Fig. 6c and d. These results fully demonstrate the synergistic advantages of the multi-channel architecture in integrating sequence features (ESM2) and network features (PPI), along with its strong suitability for LLPS prediction tasks (Fig. 6).

Finally, we compared the above snLLPS and msnLLPS models with previously reported representative LLPS protein prediction models, such as PredLLPS_PSSM [18], DeePhase [19], PSpred [20], and PSPredictor [21]. As shown in Fig. 6e and f, the comparative analysis demonstrates that msnLLPS achieves superior performance across all metrics (ACC: 0.957, MCC: 0.914), outperforming both single-feature snLLPS variants [snLLPS(ESM): ACC: 0.911, MCC: 0.825; snLLPS(PPI): ACC: 0.856, MCC: 0.714] and all existing methods including PSpred-test (ACC: 0.937, MCC: 0.875), PredLLPS_PSSM (ACC: 0.893, MCC: 0.766), DeepPhase (ACC: 0.891, MCC: 0.746), and PSPredictor (ACC: 0.837, MCC: 0.650), with particularly notable advantages in precision (0.962 versus 0.943 from PSpred-test) and AUC (0.979 versus 0.937), confirming that our multi-feature integration strategy provides more comprehensive predictive capability than conventional single-feature approaches or existing tools. In these models, snLLPS, PredLLPS-PSSM, DeePhase, PSpred, and PSPredictor only use features extracted from the protein itself, while msnLLPS fuses embedding features of protein-interacting molecules, demonstrating that PPI networks can provide additional information that affects LLPS prediction.

### Using Siamese network-based liquid–liquid phase separation models predict human proteome

In this section, we used the snLLPS (ESM2–480D) model to predict LLPS proteins in the human proteome. In order to achieve higher prediction accuracy, we have designed the following prediction framework: the framework employed anchor-based contrastive learning, where 10 LLPS and 10 non-LLPS proteins were randomly sampled as anchor sets. Each test sample was evaluated against all anchors (20 pairs per sample), with final predictions derived from averaged anchor-specific similarity scores. This ensemble approach enhanced robustness against anchor selection bias while improving generalization. To compare the prediction differences between the snLLPS model based on contrastive learning and the classical machine learning model on the human whole proteome, we also selected a Random Forest with ESM2–480 features for prediction (supplementary materials). Interestingly, among the 450 proteins predicted by snLLPS but not by Random Forest as LLPS candidates, we identified several highly plausible LLPS proteins, such as gelsolin (GELS_HUMAN, P06396), which may participate in cytoskeletal phase separation through actin binding; TIMP3 (TIMP3_HUMAN, P35625), an extracellular matrix regulator potentially forming condensates via multivalent interactions; cystathionine beta-synthase (CBS_HUMAN, P35520), which might aggregate into dynamic structures as a metabolic enzyme; HSP70 (HSP76_HUMAN, P17066), a chaperone potentially promoting phase separation under stress; ribosomal protein L7 (RL7_HUMAN, P18124), possibly involved in nucleolar assembly; the AMPA receptor subunit GRIA2 (GRIA2_HUMAN, P42262), which may form phase-separated postsynaptic densities; and pyruvate carboxylase (PYC_HUMAN, P11498), potentially regulating cellular compartmentalization via metabolic enzyme clusters. The LLPS potential of these proteins warrants further experimental validation (Supplementary materials) [8, 11, 50].

## Conclusion and discussion

In this work, we establish the Siamese network LLPS models, snLLPS and msnLLPS, which can integrate the features extracted from the protein itself and the PPI networks for LLPS prediction and achieve good accuracy even in small sample sets. Then, we used two representative graph-embedding methods, Node2vec and DeepNF, to extract the abundant features of PPI networks, and compared the impact of the two methods on model performance at different feature lengths. Our work provides a new perspective and method for automatically extracting multivalent interactions between proteins that drive LLPS, as well as a flexible framework for the integration of different types of protein features, not only for LLPS prediction but also for other downstream prediction tasks.

However, this study has several limitations that warrant discussion. Firstly, our current model primarily focuses on PPIs; however, protein–nucleic acid interactions also play a crucial role in the LLPS process. To address this important aspect in future work, we propose to extend our framework by incorporating nucleic acid interaction features, including: (i) RNA/DNA-binding domain annotations from established databases (e.g. UniProt and RBPDB), (ii) nucleic acid binding propensity scores predicted by tools like catRAPID or DeepBind, and (iii) experimentally validated protein–RNA/DNA interaction networks from resources such as NPInter and POSTAR. These features could be integrated into our existing multi-channel architecture through an additional feature processing branch with adaptive weighting, similar to our current PPI feature integration strategy. This extension would allow our model to capture the full spectrum of multivalent interactions (both protein–protein and protein–nucleic acid) that drive LLPS, while maintaining the framework’s flexibility and performance advantages demonstrated in this study.

On the other hand, there are still some shortcomings in this research. Firstly, in the PPI network, there are some pathways and molecules that are relatively more important for the LLPS process. However, this article only uses general graph-embedding methods to extract PPI features and cannot specifically handle this important information. Additionally, we only extracted PPI information from the STRING database that may be incomplete. Integrating PPI data provided by affinity purification mass spectrometry may better predict liquid-phase separation proteins. These contents are the directions that need to be considered in the future.

**Key Points**

- Extracted LLPS features from the representative large-scale pretraining evolutionary scale models as the features of the protein itself, avoiding manual design.
- Automatically extracting PPI network features that are crucial for LLPS through graph embedding models, avoiding manual design and facilitating feature integration.
- Designed a multi-channel deep metric learning framework that integrates the features of proteins themselves with the external features extracted from the interacting molecules of them, and achieves good performance at small sample sizes.

## Supplementary Material

Supplementary_FigureS1_bbaf393

Supplementary_materials_bbaf393

All_LLPS_prediction_on_uniprot_human_proteome_bbaf393

LLPS_protein_set_bbaf393

snLLPS_predicted_human_LLPS_protreins_450_bbaf393

## Data Availability

The benchmark dataset, the code of two graph embedding methods, Node2vec and DeepNF, and the Siamese network models for LLPS prediction are available at: https://github.com/ispotato/SiameseNetwork_LLPS.
