# Supplementary figures and images for: Prediction of liquid-phase separation proteins using Siamese network with feature fusion
Source: Brief Bioinform. 2025 Aug 6;26(4):bbaf393. doi: 10.1093/bib/bbaf393 (PMC12342145; doi:10.1093/bib/bbaf393)

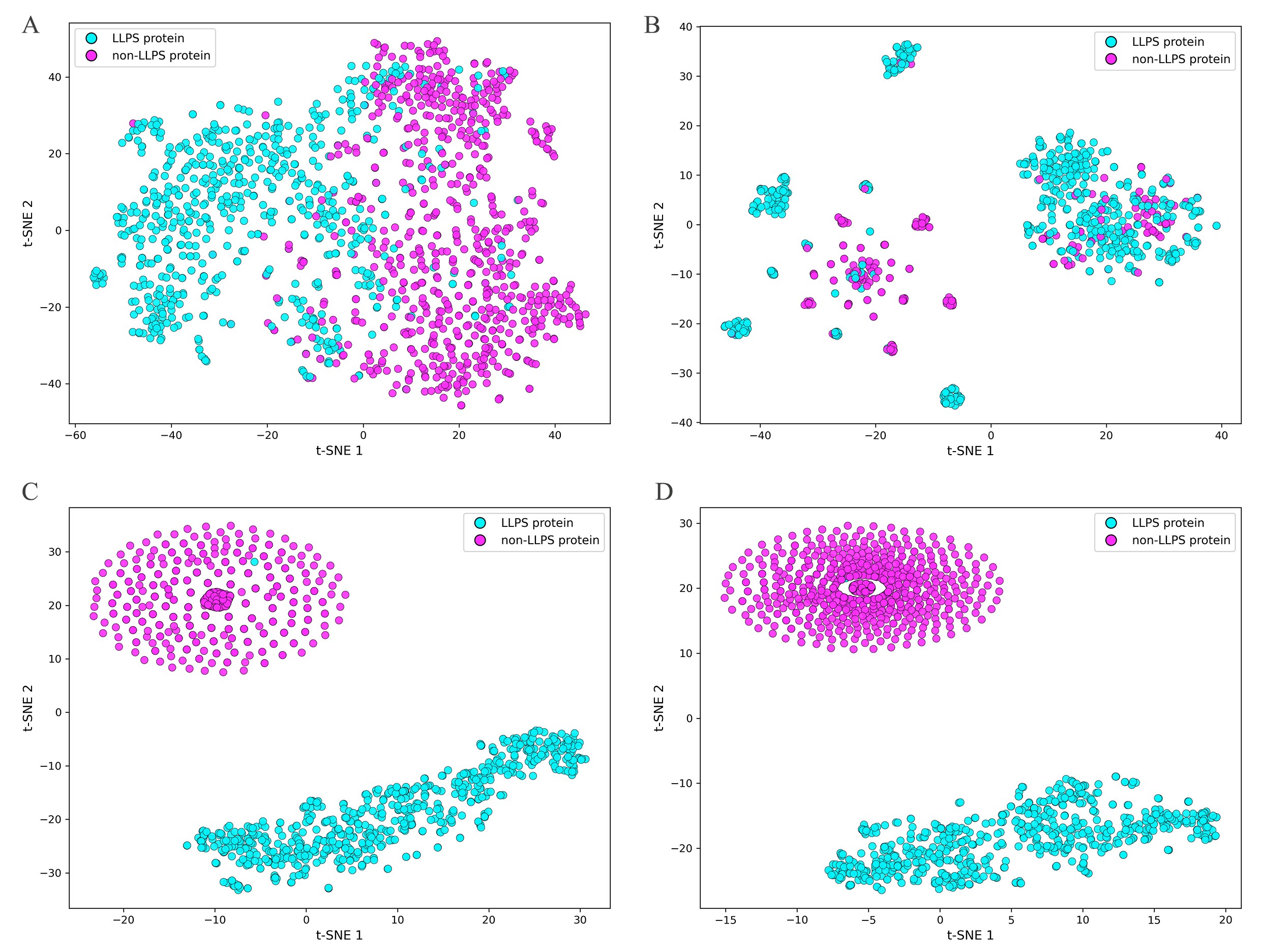

Supplement: Supplementary_FigureS1_bbaf393 [file supplementary_figures1_bbaf393.jpeg]
