# Supplementary material for: Prediction of liquid-phase separation proteins using Siamese network with feature fusion
Source: Brief Bioinform. 2025 Aug 6;26(4):bbaf393. doi: 10.1093/bib/bbaf393 (PMC12342145; doi:10.1093/bib/bbaf393)
Supplement: Supplementary_materials_bbaf393 [file supplementary_materials_bbaf393.docx]

# Supplementary materials

## 1 ProtTrans based embedding features for LLPS sample set

With the development of large language models, pre-trained protein language models (PLM) have also made significant progress [1-2]. By treating protein sequences as sentences and amino acid residues as words, PLM models based on transformer architecture can effectively capture and express complex contextual features of protein sequences, atomic level structural features and evolutionary information contained in proteins [3-7]. Representative PLM models include ESM-2, ESM-1b, ESM-1v, ProtBert, and Prot-T5-XL, among them ESM-2 performs better than previous ESM models and other PLMs in a range of structural prediction [3,7]. Using ESM2 to automatically extract protein features avoids the tedious workload and potential bias caused by manual feature design, and has achieved good results in some downstream prediction tasks [5,6]. Considering to the size of the LLPS dataset, we selected models with parameters of 8M, 35M, 150M, and 650M, corresponding to embedding feature dimensions of 320, 480, 640, and 1280, respectively [3].

The ProtTrans family of models (including ProtBert, ProtT5-XL, etc.) represents another widely adopted PLM framework. These models employ strategies similar to natural language processing models (BERT and T5 architectures) and undergo pretraining on ultra-large-scale datasets [8,9]. Through sequence reconstruction-based pretraining strategies, these models have demonstrated outstanding performance in various functional prediction and remote homology detection tasks [8,9]. To compare the impact of different large language models on LLPS prediction performance, we utilized the ProtBert submodel from the ProtTrans series. We extracted features for LLPS sample proteins under two different pretraining dataset conditions: UniRef100 (remove redundant UniProt reference sequences) and BFD (Big Fantastic Database). In both cases, the resulting embedded feature dimensions were 1024. The features of LLPS protein samples extracted using ProtTrans are detailed in the Supplementary Materials.

During our attempts to train the snLLPS and msnLLPS models using ProtTrans-derived features, we observed an unusual phenomenon: even without training, the models achieved near-perfect accuracy (~1.0), which is clearly unreasonable. To investigate this, we performed t-SNE visualization on the ProtTrans embeddings of LLPS and non-LLPS proteins (Supplementary Fig. 1), which revealed a stark separation between the two classes, explaining why the models converged without training [10]. We attribute this to the fact that our non-LLPS samples were derived from structured PDB proteins, which exhibit fundamental biophysical differences (e.g., order vs. disorder) from LLPS-prone proteins. For comparison, we also visualized the ESM2 and PPI network embeddings using t-SNE (Supplementary Figs. 2 & 3). Based on these analyses, we conclude that ProtTrans features are less suitable for LLPS prediction under the current dataset conditions because their strong bias toward capturing structural order/disorder distinctions prevents the model from learning other LLPS-relevant features. In contrast, ESM2 demonstrates superior performance in capturing finer functional characteristics critical for LLPS prediction, particularly in distinguishing phase-separation-prone proteins from other intrinsically disordered proteins (IDPs) that do not undergo LLPS.

## Reference

1. Touvron H, Lavril T, Izacard G, Martinet X, et al. Llama: open and efficient foundation language models. arXiv preprint. 2023. [arXiv:2302.13971](https://arxiv.org/abs/2302.13971arXiv:2302.13971).
2. Radford A., Wu J., Child R., Luan D., Amodei D., Sutskever I. Language models are unsupervised multitask learners. OpenAI Blog. 2019;1:9.
3. Lin Z, Akin H, Rao R, et al. Evolutionary-scale prediction of atomic level protein structure with a language model. Science. 2023;**379**:1123–30.
4. Sarumi OA, Heider D. Large language models and their applications in bioinformatics. Comput Struct Biotechnol J. 2024;**23**:3498-3505.
5. Luo Z, Wang R, Sun Y, Liu J, et al. Interpretable feature extraction and dimensionality reduction in ESM2 for protein localization prediction. Brief Bioinform. 2024; **25**:bbad534.
6. Thumuluri V, Almagro AJJ, Johansen AR, et al. DeepLoc 2.0: multi-label subcellular localization prediction using protein language models. Nucleic Acids Res. 2022;**50**:228-234.
7. Elnaggar A, Heinzinger M, Dallago C, et al. ProtTrans: toward understanding the language of life through self-supervised learning. IEEE Trans Pattern Anal Mach Intell 2022;**44**:7112–7127.
8. Elnaggar A, Heinzinger M, Dallago C, et al. ProtTrans: Towards cracking the language of life's code through self-supervised deep learning and high performance computing, IEEE Trans Pattern Anal Mach Intell. 2021;43(10):3637-3650.
9. Brandes N, Ofer D, Peleg Y, et al. ProteinBERT: A universal deep learning model of protein sequence and function, Nat Commun. 2022;13:4348.
10. Van der Maaten L, Hinton G. Visualizing data using t-SNE. J Mach Learn Res. 2008;9:2579-2605.
